# Satellite Internet and the Disruption of Telecommunications Infrastructures in Cameroon

**DOI:** 10.1177/03063127251395006

**Published:** 2025-12-26

**Authors:** Georges Macaire Eyenga

**Affiliations:** 1 Wits Institute For Social and Economic Research (WiSER), University of Witwatersrand, Johannesburg, South Africa

**Keywords:** internet, satellites, telecommunications, infrastructures, Cameroon, disruption

## Abstract

This article examines how Starlink, developed by SpaceX, constitutes a disruptive innovation in the telecommunications sector in Cameroon, where it offers connectivity in areas poorly served by traditional networks. However, there is a tension between the ideal and the market realities of democratizing internet access. On one hand, like philanthrocapitalist initiatives, Starlink promotes itself through humanitarian efforts that promise global internet access, presented as a public good. On the other hand, its business strategies privatize access and make it unaffordable for a large portion of the global population. Starlink is reshaping geopolitical and economic relations not only by competing with national operators and challenging regulatory agencies, but also by cooperating with local telecom actors and, in some cases, relying on their infrastructure to ensure the continuity of its services. Its success is further supported by a widespread crisis of user mistrust in traditional telecommunications infrastructures, fueled by frequent outages and high costs, positioning Starlink as a trust infrastructure. Our analysis shows that, while Starlink claims to promote digital inclusion, its market-driven logic deepens existing inequalities—amplifying what has long been a major challenge of internet access and rendering it even more visible and politically salient in the 21st century.

As of 2025, securing stable and continuous internet access remains a significant challenge in Cameroon, in stark contrast to narratives celebrating rapid digital transformation. Aggregate connectivity statistics (International Telecommunication Union [ITU], 2024) often obscure the everyday constraints users face. In this country, for example, residing in Dschang—a city 345 kilometers from Yaoundé with 130,000 people—I regularly experience severe connectivity issues despite my role as a researcher requiring constant online access. A 12 GB mobile data plan valid for seven days costs 5,000 CFA francs ($8), while the national monthly minimum wage is only 43,900 CFA francs ($71). Though some residents subscribe to CAMTEL’s—the national telecommunications operator—fiber-optic service for 25,000 CFA francs ($40) per month, it is only available within 300 meters of a distribution point, excluding most households, including mine. Mobile networks like MTN and Orange are unstable, and tethered connections are unreliable. Additionally, telecom operators deploy opaque marketing strategies, with overly complex service plans that confuse many users. In this commercial context, which gives the impression of a lack of regulation by the National Telecommunications Agency (ART), operators exploit consumers through non-transparent pricing and inadequate oversight. For a secondary school teacher earning 180,000 CFA francs ($290) per month, making 20 minutes of calls daily would cost 60,000 CFA francs ($97), more than one-third of their income, excluding internet fees. Against this backdrop of high costs, limited infrastructure, and unreliable service, exploring viable alternatives becomes imperative. It is in this context that we examine the potential of Starlink to address Africa’s persistent connectivity gaps.

This article interrogates the satellite internet service offered by Starlink, which has recently been made available in several African countries, including Cameroon. Contrary to SpaceX’s claims of enhancing the resilience of telecommunications infrastructures through a last-mile logic (Croshier, 2022), Starlink may in fact undermine them. It does so by establishing a sociotechnical assemblage that bypasses national regulations and consolidates the influence of private magnates. Due to its high cost and technical requirements, its access remains limited to affluent populations, excluding those it ostensibly aims to connect. This observation revives debates on universal access to the internet as a global public good (Canazza, 2018) and questions the extent to which such technology contributes to struggles for social justice, human rights, and freedom—within a broader context of inequality and the structural violence of technocapitalism (Atkinson, 2015; Milanovic, 2016; Rottenburg & Riedke, 2024; Toyama, 2015). These issues are especially salient as internet governance infrastructures are now recognized as key sites of economic and political power, increasingly appropriated for purposes far beyond their original technical and regulatory functions (Musiani et al., 2016).

This analysis draws on the theory of disruptive innovation to examine how emerging technologies such as Starlink destabilize established markets by offering efficient and more accessible services, thereby displacing dominant paradigms through a process of creative destruction (Christensen, 1997; Schuelke-Leech, 2018; Schumpeter, 1994). In Africa, where sectors like health, finance, and agriculture have already been transformed by digital platforms, mobile money, and AI, such disruptions are particularly consequential (Arthur et al., 2020). Yet, as Rottenburg (2021) suggests, disruption often emerges when prevailing belief systems collide with institutional frameworks and technical infrastructures. Building on this perspective, this reflection offers a critical theory of technoscience in the postcolonial Cameroonian context, highlighting how Starlink, despite its humanitarian discourse, reinforces capitalist dynamics of domination within a telecommunications sector largely fragmented and privatized by global tech corporations. More than a technological breakthrough, Starlink exemplifies what Allen (2016) conceptualizes as ‘topological power’—a form of spatialized influence that operates through distributed, discontinuous, and remote configurations of control. This mode of governance transcends territorial boundaries by reorganizing proximity and distance through satellite-based infrastructures and algorithmic regulation. In doing so, Starlink not only reshapes national political and regulatory landscapes, but also contributes to a broader technopolitical regime, akin to what Durand (2020) describes as ‘techno-feudalism’, where infrastructure control, rather than formal sovereignty, becomes the central vector of power. Nevertheless, this study demonstrates that Starlink’s topological power does not rely solely on satellite technologies, as its promotional discourse often suggests, but is also anchored in terrestrial infrastructures, most notably through the multiplication of Points of Presence (PoPs), as we shall examine in greater detail.

The data used in this article draw on multiple qualitative sources. First, I conducted a systematic review of national and international press coverage of developments related to Starlink since 2019, to analyze how the technology has been promoted and how it has been perceived by both African governments and users. This was complemented by semi-structured interviews with experts from national telecommunications regulatory agencies and scholars working on technoscience in Africa, as well as ten Starlink users in Cameroon and various other African countries. As part of this research, I visited one of the largest data centers on the African continent in July 2025, located in South Africa and operated by a subsidiary of a major U.S.-based infrastructure company. This facility hosts servers for global tech companies such as Google, Amazon, and Microsoft, and ensures optimal local connectivity through its management of NAPAfrica, a leading internet exchange point in the region. By facilitating content caching, direct access to cloud services, and regional peering, the data center plays a strategic role in strengthening the performance of digital services and supporting the African internet ecosystem. During my visit, I observed that the facility provides Starlink with essential physical infrastructure and interconnection capabilities, which are critical for the deployment and optimization of its satellite internet services across Southern Africa. Additionally, a netnographic approach was employed to observe and analyze daily conversations, forum discussions, and user-generated content related to Starlink on platforms such as Facebook, Twitter (X), Instagram, Telegram, WhatsApp, and LinkedIn. This online immersion offered valuable insight into the lived experiences of Starlink users across various regions of the world and brought to light the technical difficulties and contextual challenges associated with the technology’s deployment and adoption.

This analysis unfolds across five key dimensions. It begins by tracing Starlink’s emergence as a global satellite internet provider within broader transformations in digital infrastructure. It then examines the humanitarian discourse surrounding its expansion, focusing on the entanglement of philanthrocapitalism and informal rationalities. The third dimension draws on the concept of topological power to explore how Starlink reconfigures spatial relations and challenges state sovereignty. The fourth considers the extent to which Starlink contributes to the marginalization or downgrading of traditional telecommunications infrastructures. The final section investigates how trust is constructed and contested in the provision of satellite internet, particularly in regions marked by uneven digital development.

## Understanding the Emergence of Starlink

A new chapter in the history of telecommunications infrastructure is being written by SpaceX, which launched the ‘Starlink project’ in 2015. This project comprises a vast infrastructure made up of a constellation of Low Earth Orbit (LEO) satellites, Falcon9 rocket launchers, and ground-based antennas, capable of providing high speed internet worldwide, including in remote or isolated areas. Following the idea that another, better, world is possible through technology (Rottenburg, 2021), Starlink is presented as a technoscientific solution for the 2.7 billion people globally who still lack internet access, most of whom reside in developing countries. To understand why it is relevant to critically engage with Starlink, one must consider how it appears to diverge from earlier, ultimately unsuccessful attempts to connect unserved regions to the internet, such as Google’s Project Loon, which aimed to provide internet access to remote areas via high-altitude balloons in the stratosphere, or the Teledesic project, which sought to build a LEO satellite constellation to deliver global broadband connectivity (Pelton & Singh, 2015). In Africa, only 40% of the population is connected, and the disparity is particularly pronounced in rural areas, where the connectivity rate is half that of urban zones (ITU, 2022). Starlink services have since evolved into marketable products, having secured the necessary legal authorizations to operate in numerous countries since 2018 (Walsh & Zargham, 2018).

Starlink’s deployment during the 2022 Ukraine conflict marked its emergence as a geopolitical actor, illustrating how sociotechnical systems can shape international relations (Abels, 2024; Lambert, 2023; Winner, 1980), with Elon Musk acting as a political disruptor in the U.S., Ukraine, and beyond. Governed by a private entity, Starlink exemplifies the shifting power dynamics between states and corporations in the satellite telecommunications sector (Rabouam, 2024). Its global business model—high-speed, low-latency internet targeting underserved areas—strengthens SpaceX’s role in internet governance and space regulation, while intensifying competition among private providers. As early as the 2000s, scholars observed that information technologies possess ‘metapower’, the capacity to reshape identities, interests, and institutions globally (Rosenau & Singh, 2002). Starlink’s deployment in Ukraine exemplifies how private actors capitalize on crises, aligning with ‘disaster capitalism’ theories that frame emergencies as market opportunities (Klein, 2007). Today’s capitalism, increasingly voracious and extractive, extends its reach across local and global contexts, seizing both material and living resources (Mbembe, 2023).

Thus, Starlink has posed real and potential challenges to existing sovereignties. The challenges have been noted even by the research community, where scientists are concerned that the launch of tens of thousands of communication satellites into orbit could disrupt astronomical observations (see Witze, 2019, 2020). Scientific societies quickly engaged SpaceX to ensure Starlink would not interfere with radio astronomy (Lagadec, 2021). Physicist and cosmologist Marc Lachièze-Rey calls for the urgent regulation of outer space. He expresses concern over the pollution of the night sky, both optical and electromagnetic, that hampers astronomical observations. He denounces the unchecked proliferation of Starlink communication satellites and highlights the growing issue of space debris resulting from satellite overcrowding (Besnier et al., 2023). Recent studies warn of the environmental risks of satellite mega-constellations, particularly the release of aluminum oxides during atmospheric reentry, which contribute to ozone depletion (Ferreira et al., 2024).

In Cameroon, multinational corporations, humanitarian organizations, and the local elites have begun using Starlink in a context where national telecommunications infrastructures are either poor in quality or entirely absent (Bathily, 2023). We therefore start from the premise that Starlink is only competitive in countries where telecom operators are failing, and that an average telecom operator will be more competitive than Starlink, since broadband delivered from space generally cannot compete with the speed and latency of fiber optic connections to the home (FTTH) or to the cabinet (FTTC). While Nigeria and Rwanda adopted it in 2023 with minimal controversy, elsewhere on the continent its deployment has sparked public debates, leading to formal bans by governments concerned about their sovereignty (Nalwoga, 2025). This vigilance toward emerging technologies underscores the central role that states are expected to play in formulating connectivity strategies and ensuring the resilience of digital networks, as demonstrated by a recent study on the deployment of Starlink in the Arctic (Rabouam & Delaunay, 2025).

The suspicion of regulatory agencies toward Starlink is hardly surprising, as this technology not only disrupts traditional telecommunications infrastructures, often deemed inefficient, but also challenges sovereignty by functioning as an infrastructure that limits the biopolitical capacity of the state to control and regulate how its populations connect and communicate (Foucault, 2004). This is because Starlink is a technology capable of operating before agreements, thanks to its LEO satellites, which grant it mobility to cross state borders, supplant terrestrial or maritime telecommunications infrastructures, and evade national authorities’ control. Security concerns are not common to all African countries, as seen in the South African case, where Starlink’s rejection was mainly due to the company’s refusal to comply with Black Economic Empowerment laws (Dludla & Acharya, 2025). Starlink’s current inability to legally establish operations in South Africa stems from national regulatory requirements that mandate a minimum of 30% local ownership by historically disadvantaged groups, primarily Black South Africans. While over 600 foreign companies, including major U.S. firms such as Microsoft, have complied with this policy, Elon Musk has portrayed this legal condition as racially discriminatory, claiming publicly that his company is barred from operating ‘simply because [he is] not Black’ (Ngcobo, 2025). These statements, disseminated widely through his social media platform, have exacerbated diplomatic tensions between South Africa and the United States, contributing to a broader narrative of alleged anti-white policies in South Africa, particularly around land redistribution, property rights and economic empowerment measures. The controversy has thus moved beyond regulatory compliance to become entangled in geopolitics, with figures such as U.S. President Donald Trump echoing Musk’s claims and denouncing South Africa’s racial justice policies as exclusionary. This highly politicized discourse complicates Starlink’s potential entry into the South African market and underscores the intersection of digital infrastructure, national sovereignty, and contested visions of postcolonial economic justice.

Nevertheless, a political economy is emerging around the disruptive presence of Starlink, marked by the rise of local resellers, competition among economic elites, and reconfigurations in service provision models (Dosunmu, 2025). However, while Starlink represents a disruptive innovation, it is not inherently democratic: Its accessibility is constrained by the high costs of equipment and subscriptions, the requirement for digital payment infrastructures, and the availability of stable energy, factors that exclude large segments of the African population. This raises a critical question: Can Starlink be considered a trusted infrastructure? In a context where daily life on the continent increasingly relies on digital connectivity, whether for sending money, accessing banking services, teaching, communicating, or managing databases, the need for a reliable and secure internet is paramount. Trusted technologies tend to attract users by offering stable, high-quality services, contrasting sharply with the recurrent dysfunctions of traditional infrastructures. Starlink is aware of this demand and leverages it as a form of ‘competitive advantage’ (Porter, 2023), positioning itself as a provider of dependable connectivity in regions where this has long been lacking. As this article shows, Starlink actively cultivates an image of infrastructural trustworthiness, reinforcing the functioning of critical digital systems and thereby accumulating political, economic, and strategic capital (LaForge, 2023). In doing so, it contributes to the emergence of a technopolitical regime (Gagliardone, 2016), shaping not only telecommunications reforms but also influencing the broader regimes of governance and sovereignty on the continent.

## Humanitarian Rationale, Philanthrocapitalism, and Informal Logic

Scholars argued that the humanitarian rationale signifies a new regime of governance and morality, in which humanitarian action becomes the primary response to the multifaceted crises of contemporary societies (Fassin, 2012). It enables the characterization of moral choices that are important not only for states and NGOs, but also for multinational corporations that seek to promote their social responsibility (Ferrell et al., 2017). It encompasses the policies of compassion prevalent in the Global South (Machikou, 2023), motivating companies like SpaceX to develop technological solutions that, in addition to their initial intention, promise to alleviate suffering ex-post when they seek to enter new markets. As illustrated by the global deployment of medical drones by the American company Zipline (Eyenga, 2023), the diffusion of Starlink is similarly accompanied by a narrative surrounding humanitarian intervention. The project is presented as a solution to provide internet access to unconnected individuals, often due to their precariousness, remoteness, or geographic isolation, thus theoretically contributing to the reduction of the digital divide (Tamokwe Piaptie, 2013), which is known to affect civic participation, informational poverty, and democratic development (Norris, 2001). The official Starlink website states (Starlink, 2025a):

Starlink is designed to deliver high-speed, broadband internet, even to places where access has been unreliable, too expensive, or completely unavailable ….

Without the bounds of traditional ground infrastructure, Starlink can be deployed in a matter of minutes to support emergency responders in disaster scenarios. The Starlink team is proud to support and prioritize service for emergency responders around the globe and will continue to grow this support as our coverage areas expand.

Starlink enables access to essential online services and resources for rural communities that have historically gone unserved by traditional internet service providers.

In 2020, Starlink received a promise of $885 million from the U.S. government to provide broadband internet access in rural American areas under the Rural Digital Opportunity Fund (RDOF). This promise was subsequently rescinded by the Federal Communications Commission (FCC), which concluded that the company had not demonstrated its capacity to deliver the promised service, deeming that funding these extensive networks would not represent the best use of the limited resources of the Universal Service Fund. The RDOF program aimed to allocate over $6 billion to expand broadband internet access to more than 3.4 million households across 49 states and the Northern Mariana Islands (Rainbow, 2023). Although Starlink has continued its development despite the cancellation of such funding, it remains that Elon Musk’s empire (not only) has long been sustained by billions of dollars in public subsidies, enabling it to manufacture electric cars, sell solar panels, and launch rockets into space (Hirsch, 2015).

Thus, humanitarian interventions have become an opportunity for the global expansion of capitalism, presenting a dual advantage for multinational corporations: They facilitate the rapid acceptance of emerging technologies, often without much debate, under the pretext of saving lives or improving the living conditions of populations (Rottenburg, 2009). They also obscure, under the guise of emergency aid, the actual geo-economic stakes of the enterprise, particularly in relation to the conquest of space concerning Starlink (Easto, 2017). A French newspaper explains how, at the launch of Starlink, Elon Musk positioned himself as the ‘white knight of internet access’. He offered satellite internet to a small community of Native Americans, the Hoh tribe, in Washington State, and later to emergency services in Malden, a municipality in the same state the infrastructure of which had been destroyed by wildfires in the Fall of 2020. The newspaper highlights that the humanitarian rationale behind Starlink has become more evident beyond the United States: in February 2022, it decided to offer free satellite internet to one of the islands of the Tonga archipelago, which had been cut off from the world due to the eruption of the Hunga Tonga volcano. According to the journalists, publicity around these interventions have enabled lucrative business operations for the company, resulting in a staggering increase of over 245% in subscriber numbers (Seibt, 2022). As of March 2025, less than five years after its launch, Starlink reached 5 million subscribers across 125 countries.

Africa, characterized by its vast empty skies and inadequate or even nonexistent communication infrastructures (Umlauf & Burchardt, 2022), has emerged as an ideal location for the development of Starlink. It had been stated in the early 2000s that the internet is a crucial tool for reducing the digital divide and fostering socio-economic progress in Africa, thus representing an opportunity for the continent’s development (Bonjawo, 2002). In February 2023, Starlink officially conducted its first launch on the continent in Nigeria, having obtained international gateway and internet service provider (ISP) licenses from the Nigerian Communications Commission in May 2022. Initially, access to the standard service cost $25 per month, with a one-time equipment fee of $290. Since then, as the technology has spread, these prices have fluctuated significantly. The rates are not uniform across the continent and vary according to the agreements and economic realities specific to each state (Bell, 2024). After Nigeria, in March 2023 Rwanda became the second country where Starlink deployed its services, launching a pilot program covering 500 schools (Ministry of ICT and Innovation, 2023). Other countries followed suit within a year, including Mozambique, Kenya, Malawi, Zambia, Benin, and Eswatini. By 2024, Starlink had officially obtained licenses to operate in Madagascar, Sierra Leone, Zimbabwe, South Sudan, Burundi and Tchad. In 2025, Liberia, Niger, the Democratic Republic of Congo, Somalia, and Lesotho adopted this technology in their countries. The company continues to negotiate with other countries on the continent to formalize its presence. This is indeed a formalization, as Starlink is already operating informally in many countries with no official agreement. Such pre-agreement deployment is made possible by the Starlink mobile kit, which users can transport across borders and utilize beyond the national territories registered at the time of purchase. This situation has led to the prohibition of these kits in certain countries, reflecting the ongoing struggles over control of the ‘information highways’ (Amin, 2000).

The case of Northern Sudan shows the humanitarian rationale and informal use of this technology. Since April 2023, the country has been engulfed in an internal conflict between the Sudanese Armed Forces (SAF) and the Rapid Support Forces (RSF), vying for the future leadership of the country and control of its resources. This conflict has triggered a severe humanitarian crisis, resulting in over 25 million internally displaced persons and refugees in neighboring countries. During the conflict, attacks have damaged telecommunications infrastructure, depriving many areas of access to mobile networks (Zain, MTN, and Sudani), thereby compromising efforts to provide essential services and humanitarian aid in the Darfur region and parts of Khartoum and Kordofan (International Rescue Committee, 2024). It is in this context that Starlink began to be used, until the Sudanese authorities prohibited it, prompting Elon Musk to declare that he would deactivate his services in the country by restricting roaming in jurisdictions where the company did not have a license. In response to this announcement, a coalition of 94 human rights organizations operating in Sudan urged the company to keep its infrastructure active for humanitarian reasons, warning of the catastrophic consequences of a total internet shutdown on the organization of aid for the victims of the conflict (Townsend, 2024). In June 2024, the Sudanese Communications Regulatory Agency announced on its Facebook page that after two years of negotiations, an agreement had finally been reached for the official deployment of Starlink in the country.

In his critique of the humanitarian rationale, Fassin (2012) illustrates how compassionate policies governing suffering, illness, and poverty are orchestrated by governments, humanitarian organizations, and NGOs. Our case study introduces a new actor: the philanthrocapitalist enterprise, of which Starlink serves as an example. Policies supporting the disenfranchised, the victims, the ill, the displaced, and the needy are no longer exclusively the purview of governmental, religious, or humanitarian institutions. They are also acknowledged by multinational corporations, which view conflict or disaster zones as market spaces to exploit under the pretext of corporate social responsibility (CSR). As shown by Tristl and Boeckler (2024) in their study on water dispensers in Kenya, the provision of the service, as in our study the deployment of Starlink technologies, is guided by a moral obligation to guarantee internet access based on philanthrocapitalist principles that assume the realization of this accessibility through a market-oriented approach. However, these companies do not always engage significantly in addressing the inequalities from which the beneficiaries of their interventions suffer. In the case of Starlink, this reflects a reality characteristic of networked societies (Castells, 1996), where telecommunications infrastructures provide opportunities for connectivity and progress while simultaneously amplifying economic and social inequalities. From this perspective, the humanitarian rationale is transformed into a commercial rationale, thereby converting theaters of war or suffering into venues for marketing, showcasing, and experimenting with new products and infrastructures, which I describe in the following section, furthering our understanding of their disruptive effects.

## On the Topological Power of Starlink

Topological power describes the ability of a capitalist enterprise to insert itself into the fabric of everyday life and exert influence while maintaining physical distance. It captures a world in which spatial separation no longer determines proximity or remoteness, a world where distance and closeness intertwine, disrupting and reshaping spatial and temporal relations (Allen, 2011). This dynamic is vividly illustrated by the deployment of Starlink across Africa. To grasp the topological power embedded in Starlink’s presence in the telecommunications landscape, it is necessary to understand its role in constituting a new technological assemblage, a configuration in which infrastructures, standards, and protocols governing internet access are produced, negotiated, and enforced. A technological assemblage is not confined to physical infrastructure alone; it includes the regulatory, economic, and social relations through which technologies operate and gain legitimacy. It reveals how technological systems reconfigure global flows, institutional frameworks, and local practices, thereby reshaping the geographies of governance, identity, and authority (Barry, 2001).

Although Starlink’s technological assemblage remains in flux, as its use continues to be negotiated across specific national contexts, its infrastructure has been designed with global extension in mind. This has led to a form of technoscientific isomorphism, in which the architecture and logic of the system replicate across diverse geographies without necessarily aligning with existing political or regulatory boundaries (Rottenburg et al., 2024). However, this isomorphism remains relative: Starlink’s functionality, accessibility, and integration are uneven, varying significantly depending on local conditions, regulatory environments, and socio-technical practices. In this sense, Starlink exemplifies a form of topological power: it transcends spatial constraints, modulates proximity and distance, and inserts itself into distinct socio-political environments through a flexible yet cohesive infrastructural logic. Its presence is not determined by co-location but by the intensity and configuration of its connections, reshaping what it means to be ‘present’ or ‘distant’ in the digital age.

A new socio-technical configuration emerging around Starlink has implications in terms of governance, international relations, and global sociopolitical dynamics, thereby playing a crucial role in the African technoscape. By mobilizing a constellation of satellites to provide global internet access, Starlink creates a space in which various technologies, technical standards, and regulations are implemented. This space is characterized by the production and distribution of advanced telecommunications equipment, including domestic satellite dishes, wireless communication protocols, Wi-Fi routers, mounting brackets, cables, power supplies, and a mobile application (Starlink, 2025b). The deployment of Starlink requires compliance with international regulations, akin to the rules set forth by the International Telecommunication Union (ITU) regarding the allocation of radio frequencies and orbits, as well as the 1967 Outer Space Treaty (Christol, 1982), which imposes obligations on satellite operators concerning space contamination and liability for damages caused by space technologies. Starlink must also adhere to national regulations, such as those established by the Federal Communications Commission (FCC) in the United States and the telecommunications regulatory agencies of various states. This compliance work involves a range of public and private actors—engineers, scientists, regulators, and a cadre of lobbyists—collaborating to establish and enforce the technological standards necessary for Starlink to operate beyond national borders. This is because new technologies are inherently rooted in technological networks and must comply with existing standards. These innovations often face a dilemma: They need to integrate into a given technological network to gain recognition for their promises, while also needing to offer something genuinely new that disrupts established practices (Rottenburg & Riedke, 2024).

This technological assemblage has the potential to transform entire industries and render established strategies obsolete. It also demonstrates that, as an emerging technology, Starlink does not merely disrupt traditional telecommunications companies with stabilized technologies; it compels them to adjust to integrate into the newly forming market, under the threat of being surpassed. But in this way the challenges faced by established companies are rooted in technological uncertainties, ambiguous market signals, and nascent competitive structures. In such a context, emerging technologies often destroy competencies by rendering slowly acquired knowledge and assets—essential for mastering the established technology being replaced—obsolete (Day & Schoemaker, 2000). In terms of accessibility, Starlink provides internet connectivity in remote and underserved areas where fiber optic cables and cellular towers are absent or underdeveloped. This focus on rural areas (especially on the islands) aligns with the last-mile logic, which describes humanitarian projects aimed at delivering essential services to all populations, including those living in the remote territories of urban zones (McCoy, 2013). Such territories are sometimes referred to as ‘empty social spaces’ (Badié, 1993), characterized by the withdrawal of the state in favor of private initiatives, or simply as ‘hinterlands’ (Nathan, 2023), marked by the scarcity of state presence. In this context, Starlink embodies the image of a humanitarian project aimed at eliminating the ‘white zones’ where traditional services are of poor quality or non-existent.

In terms of architecture, unlike traditional technologies such as fiber optics, cellular telecommunications towers, and conventional telephone lines (ADSL), Starlink employs LEO satellites. This approach reduces reliance on local physical infrastructure, which is often constrained in regions with complex terrain, such as mountainous areas in Cameroon. This does not imply that Starlink provides a simplified infrastructure; rather, it constructs a less cumbersome system on the ground that is easier to install compared to traditional systems. Responding to the ‘kit’ logic (Lakoff & Collier, 2008), this technology offers flexibility and adaptability, particularly useful during user mobility or emergency situations. The most complex aspects of its installation involve securing an antenna at a height (preferably on the roof) and routing the provided cable to a router located inside the building. Once these steps are completed, users simply need to download the Starlink app on their smartphones and follow the instructions. The configuration phase takes approximately 20 minutes if the user encounters no major difficulties.

However, the motorized antenna, which automatically adjusts to find the optimal position, imposes certain ‘prescriptions’ (Akrich, 1992) on the user, as it must be installed in an unobstructed space. This restriction makes installation impossible in locations where direct access to the sky is limited. Once the device is secured, the connection is established within minutes, and users can then connect their devices to the Starlink Wi-Fi network, which covers up to 185 m². Thus, it is important to note that the kit’s logic, which provides transparency in using a technology—meaning it doesn’t require reinvention or assembly for each task—obscures how the infrastructure functions, leaving it a mystery to users. This invisible reality of the infrastructure becomes apparent when an unexpected event occurs, such as a technical failure (Star, 1999). Furthermore, commentators have argued that the fact that these kits cannot be repaired by local engineers in the countries where they are deployed illustrates that Starlink embodies a form of extractive technology, an insidious form of technological colonialism that contributes little to the local economy and operates as a black box (Song, 2023) in the Actor-Network Theory sense.

This architecture, which makes the technology easy to use, mobile, and not requiring in-depth technical skills from the user, aligns with the front-end logic in web development, where platforms are designed to respond directly and quickly to user requests without requiring them to concern themselves with what happens in the background (back-end): Users simply need to press a button or click to obtain a service (Umlauf & Burchardt, 2022). Tristl and Boeckler (2024) also make this point when they explain how the interface of the water dispenser machine in Kenya was designed intuitively, in such a way that it would be as easy to use for adults, as for children. However, the discretion of Starlink’s terrestrial infrastructures sharply contrasts with the congestion generated by its thousands of satellites in space. Starlink plans to deploy a constellation of 12,000 satellites, and it does not stop there: Although the initial constellation launched 4,425 satellites, on 25 October 2023, the company requested permission to launch an additional 30,000 satellites, thereby reinforcing concerns raised by astronomers. Currently, there are more Starlink satellites in orbit than all other satellites combined (Song, 2023).

This situation highlights why a critical theory of technology underscores the dependence of modern digital technologies on large-scale material and logistical infrastructures, such as cables, data centers, and various other equipment. Despite the seemingly immaterial nature of digital content, its existence relies on a highly organized and labor-intensive system of tasks, like Taylorist methods. While this process is designed for precision and efficiency, it also brings harmful consequences for human health and the environment. One example is the threat posed by falling space objects that can injure people on Earth, such as the incident in Kenya in 2024, where a piece of debris from a suspected rocket struck a residential area (Thompson, 2025), or the fall of a defunct Russian spacecraft from several decades ago (Gorman, 2025). These include issues like occupational diseases, the exploitation of workers in the electronics industry, and the suffering caused by the production chain that sustains digital technologies (Mbembe, 2023).

Another argument concerns the performance and costs of the technology. Starlink emphasizes that its satellites provide high connection speeds and low latency, asserting that most satellite internet services rely on single geostationary satellites (GEO) orbiting at an altitude of 35,786 km. This significant distance results in high latency for data transfer, making it challenging to support streaming, online gaming, video calls, and other high-bandwidth activities. To address this issue, Starlink offers a constellation of LEO satellites, situated approximately 550 km above the Earth, which significantly reduces latency due to their proximity, decreasing it from around 600 ms to just 25 ms. To recall, LEO satellites operate at altitudes ranging from 350 to 1,000 kilometers, with orbital periods on the order of several hundred minutes. Networks based on LEO satellite constellations exhibit strong resilience properties: They remain operational even in the event of localized disruptions, natural disasters, or the destruction of terrestrial infrastructure. Furthermore, the distributed, mobile, and reconfigurable nature of LEO constellations makes them significantly more difficult to physically disable compared to ground stations or geostationary satellites (Baccelli, 2024). Although Starlink vastly outperforms ADSL (Asymmetric Digital Subscriber Line) technologies that use copper telephone lines, it still struggles to compete with fiber optic connections provided by traditional telecommunications systems. This explains why Starlink can only remain competitive in contexts where traditional telecom operators are underperforming. Once these operators reach above-average performance levels, Starlink is confined to serving only a niche market.

However, one of Starlink’s comparative advantages lies in the competitive prices it offers in regions where alternatives are scarce and expensive. It is precisely here that the company’s business model accentuates inequalities. In 2023, monthly subscription fees for Starlink ranged from $25 to $100, while equipment costs varied between $200 and $650. Although Starlink’s prices are comparable to residential internet services in North America, the equipment costs were significantly higher. For the global majority, these rates remain unaffordable for all but a privileged minority. By 2024, while Starlink may celebrate obtaining licenses in several African countries, its commercial potential remains limited in the region. Despite the urgent need for connectivity in rural areas, few residents in these regions can afford the equipment and monthly subscription fees. For example, in 2022, approximately 40 million Nigerians lived below the national poverty line, set at 137,430 Naira per person per year, which is less than $2 per day (Song, 2023). In 2025, Nigeria has 65,000 Starlink users, representing approximately 0.03% of the country’s population.

## Towards a Downgrading of Telecommunications Infrastructures?

Despite Starlink’s innovative image in providing high-speed internet and global reach, can we really assert that this technology is replacing traditional telecommunications infrastructures? It is worth noting, first, that for some scholars, there is nothing particularly novel about Starlink, as satellite telecommunications services have been available for over thirty years and have proven useful in hard-to-reach regions, such as the middle of oceans or polar research stations (Lagadec, 2021). Starlink’s optimal connectivity zone is quite limited. Its target markets primarily consist of relatively affluent rural residents in isolated or sparsely populated areas, where major telecommunications companies deem it unprofitable to invest in terrestrial infrastructure. For users in well-served areas, fiber optic connections still offer a better value proposition, rendering Starlink comparatively expensive in this context. Additionally, Starlink is less competitive in urban environments due to existing competition and the satellites’ limited capacity to manage many subscribers. Its primary market is also under pressure, as telecom operators are gradually finding ways to expand their infrastructures into rural areas (Song, 2023).

From an innovation standpoint, the idea behind Starlink is not unique to it, as similar projects are being developed such as OneWeb, Amazon Kuiper, Telesat Lightspeed, SES O3b mPOWER, and China’s Hongyan and Hongyun Projects. Furthermore, Starlink’s technologies do not radically diverge from established methods of internet access provision, which combine the use of satellites and ground stations for functionality. These ground-based facilities are known as points of presence (or PoPs), an infrastructure that receives user traffic through interconnected ground stations capable of communicating with satellites. If a PoP is too distant or out of range of a Starlink satellite, the data must travel a longer distance, which in turn reduces connection speed (Kan, 2025). Starlink currently operates approximately 150 ground stations, with only a few in Africa: in Ikire and Lekki (Nigeria), Accra (Ghana), and Nairobi (Kenya). These stations are credited with having a significant impact on connectivity, as evidenced by the Nairobi station: Starlink users in Africa have begun experiencing latency of around 30 milliseconds—a drastic improvement compared with the 100 to 200 ms they previously had to work with (Kan, 2025). Moreover, a data center representative with whom I spoke noted that Starlink has recently achieved a drastic reduction in latency, from 300 ms to 20 ms, thanks to its ground stations in Botswana and Mozambique, a PoP at one data center in Johannesburg, and two internet exchange points (IXPs), NAPAfrica and JINX. According to an interviewed data center representative, the fiber-optic backbone supporting this connectivity is provided by WIOCC, MTN, and Liquid.

Starlink ultimately represents a hybrid or even creole technology that draws heavily from existing technologies. This challenges its perceived novelty. In discussions about Starlink, this allows for a shift from an innovation-focused narrative—often framed as universal but confined to specific locales—to a user-centered narrative that distinguishes between less critical innovative technologies and the underrepresented yet essential traditional technologies that play an unparalleled role in everyday life. In this user-centered narrative, technologies emerge, fade, and re-emerge, intertwining over centuries (Edgerton, 2006). Starlink is a relevant example, being an innovation that increasingly attracts media attention at the expense of ADSL and fiber optics, sometimes due to its alleged ability to overcome spatial constraints. However, it still occupies a marginal role in the daily connectivity of millions of internet users worldwide. This also implies that Starlink is not yet a central and significant technology. A user-centered narrative opens the possibility of examining the diverse ways in which Starlink becomes used, or not, across various locales and cultures globally, from the United States to urban and rural areas in Africa and Asia. This approach encourages a focus on traditional telecommunications infrastructures and the implications of Starlink’s introduction, thus shifting our perspective from the new to the old.

The evolution of Starlink echoes the dynamics observed during the introduction of satellite television in the 1990s, which marked the end of state monopolies on television broadcasting and the erosion of governments’ direct control mechanisms over information. In Africa, the emergence of transnational channels and satellite television escaped the regulation of national authorities, leading to a notable decrease in censorship. This decade constituted a decisive turning point for the African audiovisual landscape, although the political and sociocultural repercussions of these transformations were not fully understood at the time. Regions and cities once perceived as isolated became connected through satellite broadcasting technologies, thereby enhancing the ability of Africans to engage with international events and select new sociocultural reference points, reinforcing the influence of images from the sky (Ba, 1996; Dioh, 2009).

The advent of Starlink in the telecommunications sector marks a significant shift in the competitive landscape for internet service provision, echoing the disruptions introduced by satellite broadcasting in the 1990s. By enabling global internet access through its satellite constellation, Starlink challenges the national and regional monopolies traditionally held by incumbent providers. This technological intervention not only accelerates competition but also introduces a new economic model characterized by enhanced flexibility, rapid deployment, and reduced dependency on terrestrial infrastructure. However, the notion of increased competition must be nuanced. While Starlink’s presence pressures existing operators to innovate or adjust pricing strategies, it has also given rise to unforeseen forms of collaboration. Notably, the recent partnership between Starlink and the African telecom giant Airtel illustrates that the company’s market entry does not necessarily entail direct confrontation with established actors (Onukwue, 2025). Instead, it opens the door to reconfigurable alliances that reshape the organization of service delivery in complex ways. Crucially, Starlink’s capacity to operate largely outside national regulatory frameworks, by functioning independently of domestic infrastructures, has raised concerns in several countries. Telecommunications remain a highly sensitive domain of state sovereignty, and the platform’s transnational reach introduces new challenges to regulatory control, infrastructural governance, and digital sovereignty. Starlink’s arrival simultaneously disrupts existing infrastructures, diversifies the telecommunications ecosystem, and redefines the boundaries of state authority in the digital age.

In Africa, Starlink has so far established agreements with only about a dozen countries, while negotiations with a few other governments are still ongoing. However, the deployment of its technology in these countries without formal agreements has incited the ire of certain governments, which have swiftly prohibited its use by adopting repressive policies against the importation and utilization of its kits. Regulatory agencies in Mali (Houngbadji, 2024), Côte d’Ivoire (Maryelle, 2024), Burkina Faso (Zogo, 2024), Democratic Republic of Congo (before 2025) (Abely, 2024), Zimbabwe (Kabweza, 2024), South Africa (Sehloho, 2024), Chad (Tchadinfos, 2023), Senegal (Sall, 2023), and Cameroon (Koné, 2024) have demanded that the company regularize its services and comply with national telecommunications requirements or with the rules concerning foreign investments, specifically the case for South Africa. These agencies contend that the illegal operation of Starlink poses a threat to national security and fair competition. Moreover, the absence of physical infrastructures or an official presence of the company in these countries (except for Nigeria) complicates the ability of these agencies to exert control over Starlink’s services. This situation also makes it difficult for governments to hold the company accountable for the content transmitted via its signals. From a financial perspective, the situation is also considered to be problematic, as Starlink’s activities generate revenue without the company paying taxes to the public treasuries of countries with which no agreements have been established. This issue is exacerbated by the fact that Starlink minimizes its investments in the countries where it operates.

In Southern Africa, Starlink has been banned in many countries, except for Zambia. In Zimbabwe, the government initially prohibited Starlink, leading to the arrest of several private traders and some elite family members who were illegally using the service (Kudzayi Mutisi, 2024), even as the national broadcaster used it. It was not until May 2024 that an agreement was finally reached to regularize the service. However, these agreements, which influence the telecommunications economy, have been marred by patronage. For example, Zimbabwe’s President Emmerson Mnangagwa granted a license to Starlink after the company agreed to an exclusive partnership with a telecommunications firm owned by one of his friends, Wicknell Chivayo. This exclusivity sparked outrage among several elites and members of the ruling ZANU-PF party, ultimately leading to its cancellation by the postal and telecommunications regulatory agency (Quadri, 2024).

In some cases, repressive measures have been implemented to ensure that the service cease to operate in the absence of formal agreements. In Cameroon, for instance, a notice issued by the Directorate General of Customs on 15 April 2024, titled ‘Ban on the Importation of Starlink Kits’, was sent to various heads of customs sectors (Koné, 2024). This notice emphasizes that the uncontrolled use of these devices could compromise national security and reminds that the importation of telecommunications transmission equipment in Cameroon is subject to prior approval from the Telecommunications Regulatory Agency (ART). It also instructs customs agents to systematically seize any telecommunications equipment imported across Cameroonian borders as part of the fight against the illegal use of Starlink. This concern is not without basis. In a recent report, the French newspaper *Le Monde* underscored that Starlink is being used by armed groups in Africa to facilitate communication in regions beyond the reach of conventional terrestrial communication infrastructures (Cuordifede, 2025).

In response to this ban, Starlink revised its terms of service, stating that the availability of its service would now depend on factors that included regulatory approvals from the countries where it operates. While announcing that it had begun negotiations with several governments to comply with local regulations, the company also declared that its service would be suspended as of 30 April 2024 in countries where it did not have agreements (Djoyum, 2024). In practice, however, this suspension announcement was never enforced, and Starlink continued to operate discreetly throughout the country. My investigations in Cameroon revealed that people continued to use the technology informally by registering their devices in other countries where the technology was authorized, although at a higher cost than if the devices had been registered in the user’s home country. Furthermore, the ‘Mobile-Regional plans’—allowing a user to move from one country to another while still using the service—were limited to a duration of two months. After this period, the user would need to either to change the country associated with their account or return to the country where the service was originally ordered, under penalty of having their access suspended.

## Trust in Satellite Internet

In March 2024, sub-Saharan African countries experienced a massive internet outage caused by the failure of several undersea telecommunications cables, including the West Africa Cable System (WACS), the Africa Coast to Europe (ACE), and MainOne. These infrastructures are crucially as they carry most of the global internet traffic, with one of these cables, spanning 15,000 km, connecting Portugal to Cape Town, South Africa (Africanews and Agences, 2024). These terrestrial networks constitute a critical link in the development of digital infrastructure in Africa, enabling the transfer of data between submarine cable landing points and the continent’s inland regions (Coelho, 2025). To mitigate the effects of this outage, Cameroon redirected internet data traffic through the South Atlantic Inter Link (SAIL) submarine cable system, linking the country to Brazil. In May 2024, East African countries also encountered similar disruptions, due to damage to the Seacom and Eassy undersea cables, which connect the region to the global network (Pecquet & Bojang, 2025). In addition to these significant outages, the daily use of the internet via mobile networks from telecom operators is regularly disrupted, leading to growing dissatisfaction among users. In September 2024, the Telecommunications Regulatory Agency (ART) in Cameroon reported a notable deterioration in the quality of mobile services from Orange, MTN, and Camtel, the three main mobile operators (2G, 3G, 4G). ART attributed this decline to several factors: an electricity shortage preventing the operation of infrastructures, fuel supply difficulties delaying technical interventions, and frequent fiber optic cable breaks disrupting data transmission (ART, 2024).

The recurrent failures of telecommunications infrastructures in Cameroon significantly undermine public services and the business environment, fueling debates about their reliability and eroding user trust. These malfunctions contribute to the perception of telecommunications systems as unreliable, fostering a broader sense of ‘distrust’ among users, a sentiment akin to structural unreliability (Carey, 2017). As interpersonal trust and mistrust in Cameroonian contexts have been shown to be shaped by experiences of uncertainty and suspicion, notably in the domain of witchcraft (Geschiere, 2013), so too is trust in technological infrastructures shaped by everyday encounters with service interruptions, fears of internet outages, surveillance, and the perceived accountability of operators. Trust in infrastructure, however, is never solely about the technology or the entity in which it is placed. As Dalinghaus (2016, p. 3) on trust suggests, the saying ‘In ___ we trust’ refers not only to personal relationships—with friends, strangers, institutions, or service providers—but also to the broader material and social arrangements through which trust is cultivated and enacted. These include overlapping technological systems, social networks, and shared normative expectations. Moreover, trust in new technologies tends to increase when these systems are embedded as one among multiple reliable channels for conducting meaningful transactions, such as the storage and transfer of value. In this sense, trust is not merely a human sentiment but a relational dynamic involving individuals, machines, infrastructures, and sociotechnical systems. As such, it remains a crucial condition for economic prosperity and social development (Dalinghaus, 2016).

The increasing technicization of the world reveals that this need for trust also pertains to interactions with the material environment, whose stability is a determining factor for optimal and predictable planning of social activities. In the scientific domain, trust among researchers is indispensable for accepting results that they cannot always themselves verify (Collins & Pinch, 2000), demonstrating that science fundamentally relies on these trust relationships (Latour, 1987). Similarly, a trusted internet (Blefari-Melazzi et al., 2011), which guarantees the interconnectivity of terminals essential for the transaction chain, is indispensable for the functioning of a digital economy. Moreover, the loss of trust in routinized sociotechnical systems, which are taken for granted, often becomes a necessary condition for innovation (Rottenburg & Riedke, 2024). This positions Starlink as a foundational trust infrastructure as it supports and interconnects all systems and infrastructures that depend on the internet. A stable internet offers assurances for the effective functioning of production, distribution, and financial market chains (Castells, 2001).

However, framing Starlink as an emerging trust infrastructure in Cameroon calls for a critical interrogation of the geopolitical, commercial, and epistemic logics that underpin this perception. From a critical standpoint, infrastructures are not neutral devices that merely facilitate connectivity; they are deeply embedded in relations of power, ownership, and control. Starlink’s promise to circumvent terrestrial vulnerabilities reconfigures dependency, shifting it away from local infrastructures toward a technosovereign, privatized, extranational actor that largely escapes public accountability mechanisms. While this transition may temporarily stabilize internet access and restore a certain level of trust in connectivity, it also entails a structural displacement of infrastructure governance, increasingly beyond the reach of state regulation. The portrayal of Starlink as a solution to Cameroon’s infrastructure crisis tends to obscure the political economy of such arrangements: Who owns the satellites? Who controls the data? Under what conditions is access guaranteed or denied? In this beclouded context, trust ceases to be a relational, socially embedded process and becomes a commodified experience, delegated to a singular global actor.

By reframing trust in infrastructure in terms of mere technical performance or reliability, there is a risk of erasing the historically situated, materially contingent, and politically contested processes through which infrastructures acquire their legitimacy. There is also a geopolitical dimension to the issue of trust on the internet, which involves strategic competition between the United States and China (Singh et al., 2025). It raises the question of whether we should ultimately rely on internet services provided by an American company—such as Starlink—or by a Chinese company—such as SpaceSail—considering the power dynamics and strategic rivalries between these two global superpowers. This calls for viewing Starlink not simply as a technological fix, but as an object of critical inquiry into how digital sovereignty, technological dependencies, and infrastructural power are being reconfigured in Africa under the guise of innovation and efficiency.

## Conclusion

In nearly a decade, Starlink, with its innovative model of providing internet via LEO satellites, has profoundly changed the vision of what the future of internet access could be. This project has quickly established itself as a leading emerging technology, prompting other companies to explore similar solutions. This new race for space, driven by the promise of global internet access, reflects the growing political importance of internet access, now regarded as a fundamental human right and a crucial lever for economic development, national sovereignty, and the fight against global inequalities. Where Starlink stands out is in its ability to leverage infrastructural gaps, particularly in Cameroon, to establish itself as a viable alternative to traditional telecommunications operators. By offering connectivity in areas poorly served by terrestrial networks, Starlink positions itself as a trust technology capable of supporting entire ecosystems that depend on reliable connectivity. However, this humanitarian promise is accompanied by more complex economic realities. Starlink and its competitors operate within a capitalist logic that, far from democratizing internet access, tends to further privatize it, rendering their services often inaccessible to a significant portion of the most vulnerable populations: There is a tension between the stated desire to democratize internet access and the reality of the commodification of this resource.

The global deployment of satellite internet, while reshaping communication infrastructures, introduces a new form of topological power. Companies like Starlink not only redefine the telecommunications landscape, they may also transform geopolitical relationships by competing/or collaborating with national operators and exerting direct influence on political events. In developing countries regulatory agencies find themselves overwhelmed, struggling to control a service that could accelerate access inequalities. The case of Starlink illustrates this ambivalence: a technology that presents itself as a savior but whose high costs and business practices contradict the vision of an inclusive and universal internet. The humanitarian interventions emphasized by the company often mask a strategy of commercial penetration, exploiting crisis contexts to establish itself without genuine debate about its technoscientific implications. The disruptive nature of the technology shakes traditional models, unsettling states, national operators, and regulators, imposing a new reality where internet access, rather than being a public good, becomes a luxury product. Ultimately, the deployment of Starlink aptly illustrates the contradictions of modern technological capitalism: a project that, while contributing to digital inclusion, might exacerbate disparities.
